# Key for European species of the *Cheilosia proxima* group (Diptera, Syrphidae) with a description of a new species

**DOI:** 10.3897/zookeys.269.4619

**Published:** 2013-02-15

**Authors:** Ante Vujić, Snežana Radenković, Sonja Trifunov, Tijana Nikolić

**Affiliations:** 1 Department of Biology and Ecology, Faculty of Sciences, University of Novi Sad, Trg Dositeja Obradovića 2, 21000 Novi Sad, Serbia

**Keywords:** Diptera, Syrphidae, *Cheilosia barbafacies*, *proxima* group, new species

## Abstract

A new hoverfly species, *Cheilosia barbafacies* Vujić & Radenković **sp. n.** (Diptera, Syrphidae), is described and distinguished from the closely related species *Cheilosia pascuorum* Becker, 1894, based on material collected from the mountains of the Balkan Peninsula. Diagnostic characteristics and an identification key for the members of the *proxima* group of *Cheilosia* s. str., including the new taxon, are provided.

## Introduction

*Cheilosia* Meigen, 1822 is the largest Palaearctic hoverfly (Diptera, Syrphidae) genus with nearly 300 species listed by Peck (1988), and 439 described species worldwide (Thompson et al. 2010). Its distribution extends to the Nearctic (more than 80 species), Oriental (about 50 species) and northern part of the Neotropical regions (one species from Chiapas, Mexico, and another one from Guatemala) (Ståhls et al. 2004; Thompson et al. 2010).

This genus belongs to the monophyletic tribe Rhingiini of the subfamily Eristalinae, as a sister group of Eumerini (Ståhls et al. 2003). According to Peck (1988), the tribe Rhingiini (as Cheilosiini) includes the genera *Chamaesyrphus* Mik, 1895, *Cheilosia*, *Endoiasimyia* Bigot, 1882, *Ferdinandea* Rondani, 1844, *Ischyroptera* Pokorny, 1887, *Macropelecocera* Stackelberg, 1952, *Pelecocera* Meigen, 1822, *Portevinia* Goﬀe, 1944, *Psarocheilosia* Stackelberg, 1952 and *Rhingia* Scopoli, 1763. The phylogenetic relationships of the tribe Rhingiini and the genus *Cheilosia* (Diptera, Syrphidae) were investigated by Ståhls et al. (2004) using morphological and molecular characters. The monophyly of subtribes of Rhingiini remained ambiguous, especially because of unstable phylogenetic placements of the genera *Portevinia* and *Rhingia*, while most of subgenera of *Cheilosia* appeared as monophyletic clades.

The subgeneric classiﬁcation of *Cheilosia* has been changed from Becker’s (1894) division of the genus into four artiﬁcial groups (A–D) to Barkalov’s (2002) description of 13 subgenera, of which 9 are new (*Cheilosia* Meigen, 1822; *Endoiasimyia* Bigot, 1882 (= *Sonanomyia* Shiraki, 1930); *Taeniochilosia* Oldenberg, 1916 (= *Nigrocheilosia* Shatalkin, 1975); *Hiatomyia* Shannon, 1922; *Neocheilosia* Barkalov, 1983; *Eucartosyrphus* Barkalov, 2002; *Floccocheila* Barkalov, 2002; *Pollinocheila* Barkalov, 2002; *Montanocheila* Barkalov, 2002; *Nephocheila* Barkalov, 2002; *Conicheila* Barkalov, 2002; *Convocheila* Barkalov, 2002; *Rubrocheila*, Barkalov 2002). Several of these subgenera were recognized earlier as species groups (*nigripes, longula, illustrata, alpina, velutina, scanica, sachtlebeni, formosana*) (Barkalov 1983). Before Barkalov’s subgeneric division (2002), the names of subgenera *Nigrocheilosia* Shatalkin, 1975, *Neocheilosia* Barkalov, 1983 and *Cheilosia* s.str. were also in use. The *nigripes* species group corresponds to subgenus *Nigrocheilosia*, the *scanica* species group to subgenus *Neocheilosia*, and most members of the *velutina* group to *Cheilosia* s.str. The monophyly of the genus *Cheilosia*, as well as subgenera (*Nigrocheilosia*, *Neocheilosia*, *Cheilosia*) and some species groups, were well supported by molecular analysis (Ståhls and Nyblom 2000).

All known species undergo larval development in specific plants or fungi, although some species feed on a wide range of plants. There is only one known exception, the species of the subgenus *Neocheilosia* Barkalov, 1983, which feed on sap and cambium of coniferous trees. One of the most serious pests of genus *Cheilosia*, is the species *Cheilosia vulpina* (Meigen, 1822) that infested 50% of artichoke (*Cynara scolymus*) crops in Northern France during the 1980s (Rotheray and Gilbert 2011). Although larvae of species from the *proxima* group are mostly undescribed, except *Cheilosia proxima* (Zetterstedt, 1843) and *Cheilosia vulpina*, they were observed from different plants by several authors. Very often these larvae mine stems, roots or rhizomes, or, rarely, graze roots externally, as observed in larva of *Cheilosia proxima* found on *Cirsium palustre* (Rotheray and Gilbert 2011). *Cheilosia gigantea*(Zetterstedt, 1838) was reported from *Rumex* sp.; *Cheilosia pascuorum* Becker, 1894 is an internal feeder in *Cynoglossum officinale*; *Cheilosia proxima*(Zetterstedt, 1843) was collected from *Cirsium palustre* and *Cheilosia oleraceum*; *Cheilosia rufimana***(**Becker, 1894) was observed ovipositing on *Polygonum bistorta*, while *Cheilosia velutina*Loew, 1840 mines the stems of *Cirsium palustre* andthe rhizome of *Scrophularia nodosa* (Speight 2012). While phytophagous hoverflies can cause economic damage by attacking cultivated plants, they can also be used beneficially to control weeds. Examples of this are *Cheilosia psilopthalma* (Becker, 1894) and *Cheilosia urbana* (Meigen, 1822), which have been found to be efficient biological control agents for *Hieracium* spp., and which are sufficiently host-specific for release in New Zealand where no native *Hieracium* species exist (Grosskopf et al. 2002).

These blackish hoverflies without mimetic features still cause identification troubles for taxonomists, due to the existence of many morphologically similar taxa with variable characters. There is no key through which all European species of *Cheilosia* can be identified. Recently, attempts have been made to stabilise the nomenclature of western European *Cheilosia* species, by dealing with small groups of closely related species (Barkalov and Ståhls 1997, Claussen 1998, Claussen and Speight 2007, Haarto and Kerppola 2007, Speight 2012). In the last decade, only a few species were described from Europe, including *Cheilosia ingerae* Nielsen & Claussen, 2001 (Nielsen and Claussen 2001), *Cheilosia naruska* Haarto & Kerppola, 2007 (Haarto et al. 2007) and *Cheilosia thessala* Claussen & Ståhls, 2007 (Claussen and Ståhls 2007).

Vujić (1996), in his revision of the *Cheilosia* species from the Balkan Peninsula, recorded 77 species and two subspecies: nearly half of the 175 registered European species (Speight 2012). The influence of different biogeographical regions over different geological periods resulted in great biodiversity on the Balkan Peninsula, making it one of the important speciation centres in Europe (Vujić 1996, 1997, 1999a, 1999b, Vujić et al. 1994, 2008). Many species of the genus *Cheilosia* have been described from this area, e.g. *Cheilosia alba* Vujić & Claussen, 2000 (Vujić and Claussen 2000), *Cheilosia balkana* Vujić, 1994 (Vujić 1994b), *Cheilosia bracusi* Vujić & Claussen, 1994 (Vujić and Claussen 1994b), *Cheilosia clama* Claussen & Vujić, 1995 (Claussen and Vujić 1995), *Cheilosia griseifacies* Vujić, 1994 (Vujić 1994a), *Cheilosia katara* Claussen & Vujić, 1993 (Claussen and Vujić 1993), *Cheilosia orthotricha* Vujić & Claussen, 1994 (Vujić and Claussen 1994a), *Cheilosia redi* Vujić, 1996 (Vujić 1996), and *Cheilosia vujici* Claussen & Doczkal, 1998 (Claussen and Doczkal 1998).

After detailed analysis of published material under the name *Cheilosia pascuorum* Becker, 1894 from the Balkan Peninsula (Vujić 1996), one new morphologically cryptic species was discovered and is described in the present text. This new cryptic species belongs to the *proxima* group of *Cheilosia* s.str., together with the other Palaearctic species *Cheilosia balkana*, *Cheilosia gigantea*, *Cheilosia ingerae*, *Cheilosia pascuorum*, *Cheilosia proxima*, *Cheilosia rufimana*, *Cheilosia velutina*, and *Cheilosia vulpina* (see Table 1 for distribution). Nielsen and Claussen (2001) presented an identification key and diagnostic characters for the Fennoscandian species of the *proxima* species group which is here adapted and expanded. In addition, relationships between this species group and related species are discussed.

**Table 1.** List of species of *Cheilosia* belonging to the *proxima* group and their distribution.

**Species name**	**Species distribution, from Speight (2012), with additional information for the Balkan Peninsula**
*Cheilosia balkana*	Alps (Italy), Balkans (Montenegro, Serbia, Slovenia).
*Cheilosia barbafacies* sp. n.	Dinaric mountains on the Balkan Peninsula
*Cheilosia gigantea*	Fennoscandia south to the Alps; Germany eastwards through northern and central Europe together with northern Italy and the Balkans (Slovenia, Bosnia and Herzegovina, Serbia, Montenegro, FRY Macedonia, Bulgaria) into European parts of Russia, and from Ukraine to the Caucasus; in Siberia from the Urals to the Pacific coast.
*Cheilosia ingerae*	Northern Norway, Sweden and Finland.
*Cheilosia pascuorum*	Alps (France, Germany, Switzerland, Austria), Romania, parts of European Russia, the Balkans (Serbia, Montenegro).
*Cheilosia proxima*	Fennoscandia south to the Pyrenees and the mountainous regions of Spain; Britain eastwards through much of Europe, the Balkans (Slovenia, Croatia, Bosnia and Herzegovina, Serbia, Montenegro, FRY Macedonia, Greece, Bulgaria) into Turkey and European parts of Russia; in Siberia from the Urals to Kamchatka.
*Cheilosia rufimana*	From Finland, Denmark and Belgium eastwards through mountainous regions of central Europe to the Balkans (Serbia, Bulgaria); Ukraine; Kazakstan; Asiatic Russia.
*Cheilosia velutina*	Fennoscandia south to Spain; from Ireland eastwards through much of Europe into Russia and through Siberia to the Pacific coast.
*Cheilosia vulpina*	Denmark to the Pyrenees and northern Spain; from England eastwards through central Europe to the central and southern parts of Russia as far as western Siberia.

## Methods

The characters used in the key, descriptions, and drawings employ the terminology established by McAlpine (1981); the male genitalia characters are defined by Claussen (1998) and Nielsen and Claussen (2001).

The specimens under study were collected by sweep netting. To study male genitalia, specimens were relaxed and the genitalia were extracted using an insect pin with a hooked tip.

Genitalia were cleared by boiling individually in tubes of water-diluted KOH pellets for 5 min. This was followed by brief immersion in acetic acid to neutralize the KOH and immersion in ethanol to remove the acid. Samples were stored in microvials containing glycerol. Drawings were made with an FSA 25 PE drawing tube attached to a binocular microscope. Measurements were taken with an eye piece graticule or micrometer.

All the studied material, including type material, has been deposited at the Department of Biology and Ecology, Faculty of Sciences, University of Novi Sad, Serbia (FSUNS).

## Systematics

### Tribe Rhingiini

#### Genus *Cheilosia* Meigen, 1822

#### Type species.

*Syrphus flavipes* Panzer, 1798

### Subgenus *Cheilosia* Meigen, 1822

*Chilosia* Agassiz, 1846

*Cartosyrphus* Bigot, 1883

*Chilomyia* Shannon, 1922

*Chaetochilosia* Enderlin, 1936

*Dasychilosia* Enderlin, 1936

*Proxima* species group

#### Diagnosis

**Figure 1.**
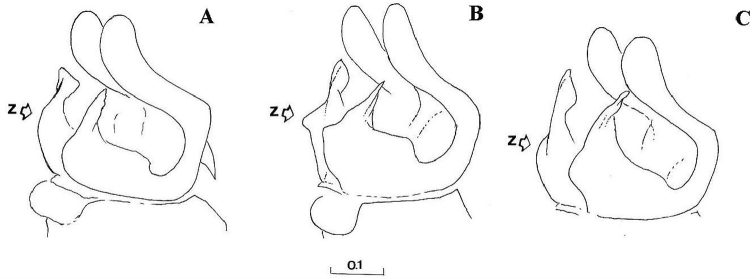
Gonostylus, dorsolateral view (z indicates the dorsal margin of gonostylus): **A** *Cheilosia barbafacies* sp. n., Durmitor, Montenegro **B** *Cheilosia pascuorum*, Doroslovo, Serbia **C** *Cheilosia pascuorum*, Kopaonik, Serbia. Scale in mm.

**Figure 2.**
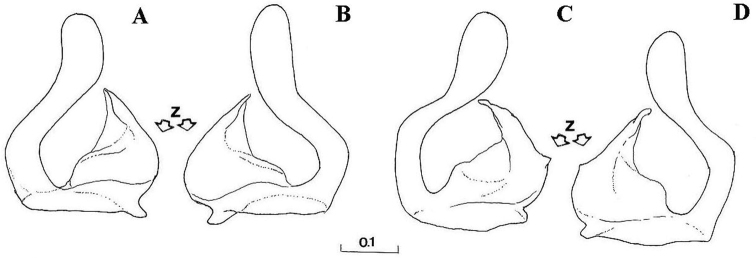
Gonostylus (z indicates the dorsal margin of gonostylus). **A–B** *Cheilosia barbafacies* sp. n.: **A** left gonostylus, left lateral view **B** right gonostylus, right lateral view **C–D** *Cheilosia pascuorum*: **C** left gonostylus, left lateral view **D** right gonostylus, right lateral view. Scale in mm.

**Figure 3.**
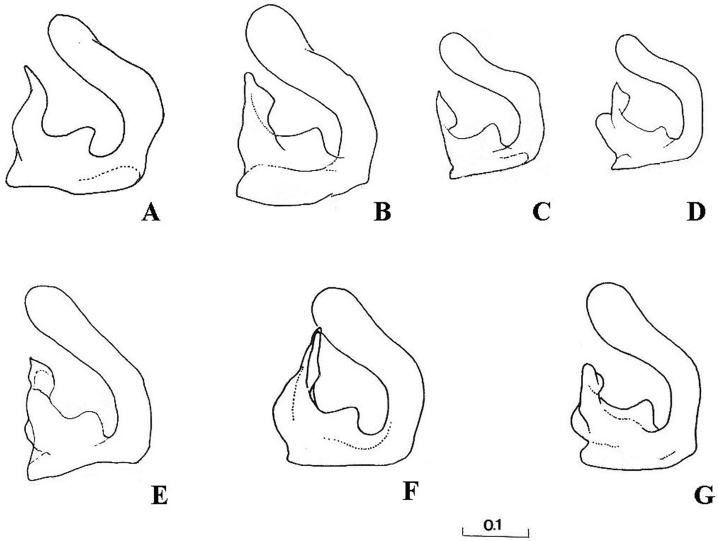
Right gonostylus, right lateral view: **A** *Cheilosia barbata* **B** *Cheilosia ingerae* **C** *Cheilosia balkana* **D** *Cheilosia proxima* **E** *Cheilosia gigantea* **F** *Cheilosia velutina* **G** *Cheilosia rufimana*. Scale in mm.

**Figure 4.**
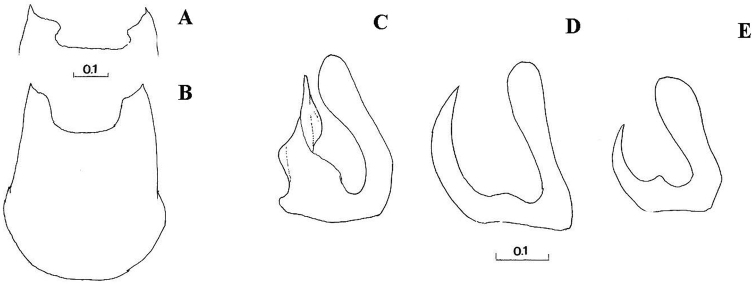
**A–B** *Cheilosia barbafacies* sp. n., theca of hypandrium, ventral view: **A** Montenegro, Durmitor **B** Bosnia-Herzegovina, Jahorina **C–E** right gonostylus, lateral view: **C** *Cheilosia vulpina* **D** *Cheilosia lasiopa* **E** *Cheilosia melanopa*. Scales in mm.

Eyes pale haired; antennal pits separated; vertex grey dusted; central prominence rounded and more protruding than lateral corner of subcranial cavity, in lateral view; face at the level of central prominence less wide than half width of head. Probasisternum of protorax not fused with adjacent sclerites; anterior anepisternum bare; scutellum with black, exceptionally yellow, marginal setae; katepisternum with upper and lower hair patches connected or narrowly divided, entirely dusted; legs predominantly black, except tibiae usually paler on both ends; front coxa without lateral tooth; last tarsomere of front leg unmodified; in females, some hairs on hind tibiae longer, at least more than half of its width. Sternites of abdomen entirely grey dusted; male genitalia: gonostylus with a characteristic dorsal lobe (Figs 1, 2), neither S-shaped (Fig. 3A) nor sickle-shaped (Fig. 4D, 4E).

**Comments.** This group is related and morphologically similar to the following *Cheilosia* s.str. species: *Cheilosia barbata* Loew, 1857, *Cheilosia naruska*, *Cheilosia aerea* Dufour, 1848, and *variabilis* group of species, i.e. *Cheilosia melanopa* (Zetterstedt, 1843), *Cheilosia redi*, *Cheilosia honesta* Rondani, 1868, *Cheilosia variabilis* (Panzer, 1798). Nevertheless, all of them can be distinguished by a combination of characters: *Cheilosia barbata* has a S-shaped gonostylus (Fig. 3A) and females have less dusted sternites, undusted central part of the katepisternum and a narrower frons with parallel sides; in *Cheilosia aerea*, hairs on the anterior anepisternum are present and sternites are less dusted in some specimens and populations; in *Cheilosia naruska*, sternites are undusted except for slightly dusted anterior and posterior margins; males in *variabilis* group have sickle-shaped gonostylus (Fig. 4D, 4E) and females have very short and adpressed hairs on hind tibiae.

#### Cheilosia barbafacies

Vujić & Radenković sp. n.

urn:lsid:zoobank.org:act:9F2ABB52-652B-4652-AD7C-8ACB4D8579C8

http://species-id.net/wiki/Cheilosia_barbafacies

Figs 1A 2A, 2B 4A, 4B 5 [Fig F6] –7 8A, 8C 9 [Fig F10] –11 12A, 12B

Cheilosia honesta of Šimić, 1987 (in part).
Cheilosia pascuorum of Vujić, 1996 (in part).

##### Type-locality.

MONTENEGRO: Durmitor, Škrčko-Sušički basen, 43˚11'7"N, 19˚3'28"E, broad-leaf forest, 25 June 1995, A. Vujić leg.

##### Type-specimen:

**Holotype** ♂, in excellent condition. MONTENEGRO. Original label: “Durmitor YU / Skakala 25.06.’95. / leg. Vujić.” 43°10'16"N; 18˚59'56"E (FSUNS 05768).

**Paratypes**, in excellent condition. MONTENEGRO**:** ♂ Original label: “007. Durmitor / Skrcka jezera / 5.07.1983.”43°8'8"N; 19°0'56"E (published in Šimić (1987) as *Cheilosia honesta*) (FSUNS 05758); ♂ Original label: “199 G. Durmitor / Luke / 8.07.1991. YU.” 43°7'37"N; 19°0'5"E (published in Vujić (1996) as *Cheilosia pascuorum*) (FSUNS 05759); ♂ Original label: “199 H. Durmitor / Luke / 9.07.1991. YU.” 43°7'37"N; 19°0'5"E (FSUNS 05763); 3♂ Original label: “199 H. Kanjon Susice / 9.07.1991. YU.”43°12'41"N; 18°59'44"E (FSUNS 05760, 05761, 05766); ♂ Original label: ”Durmitor 8.07.92. / Skrcko Zdrijelo YU / leg.Vujic. ”43°7'7"N; 19°0'53"E (FSUNS 05765); ♂ Original label: “Durmitor 2.07.93. / ka Prutasu YU / leg. Radnovic S.” 43°10'16"N; 18°59'56"E (FSUNS 05764); ♂ Original label: “Durmitor 1.6.1994 / KanjonSusice YU / leg. Vujic.” 43°12'41"N; 18°59'44"E (FSUNS 05770); ♂ Original label: “Durmitor YU / Skakala 6.7.1994. / leg. Vujic.” 43°11’16"N; 19°0'21"E(FSUNS 05767); ♂ Original label: “Durmitor 30.06.93. / Skrčko jezero YU / leg. Radnovic S.” 43°8'8"N; 19°0'56"E (FSUNS 05762); ♂ (FSUNS 05771) ♀ (FSUNS 05769) Original label: “Durmitor 25-26.5.96. / Susica-Skrke YU / leg. Vujic”. 43°11'7"N; 19°0'28"E. BOSNIA-HERZEGOVINA: ♂ Original label: “1613 Bosna / Jahorina / 14.05.1989.” 43°42'25"N; 18°34'13"E (published in Vujić (1996) as *Cheilosia pascuorum*) (FSUNS 05757).

##### Description.

MALE (Figs 1A, 2A, 2B, 4A, 4B, 5, 6C, 8A, 8C, 9A, 11A, 12A, 12B).

**Figure 5.**
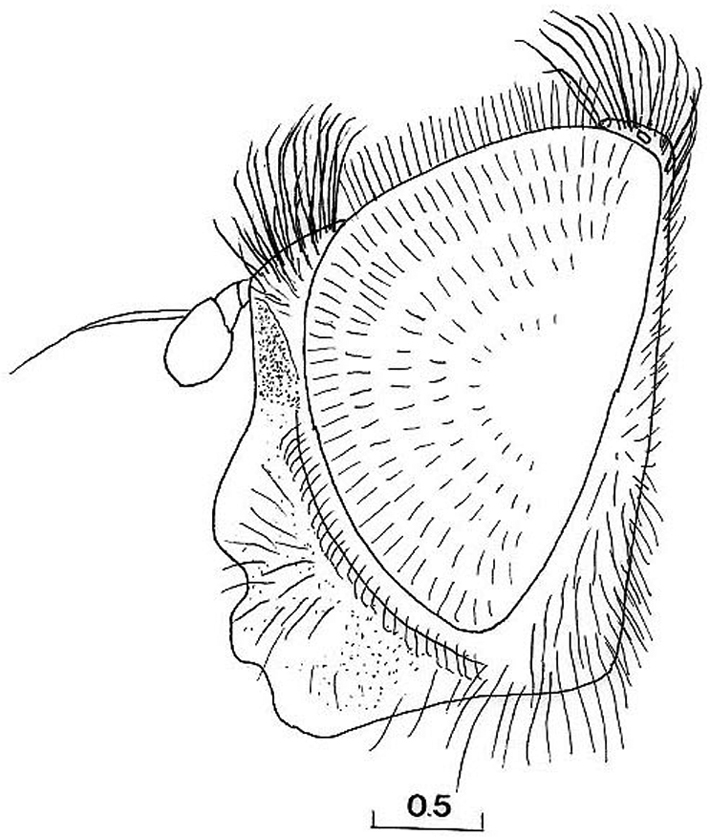
*Cheilosia barbafacies* sp. n., male, head, lateral view. Scale in mm.

**Figure 6. F6:**
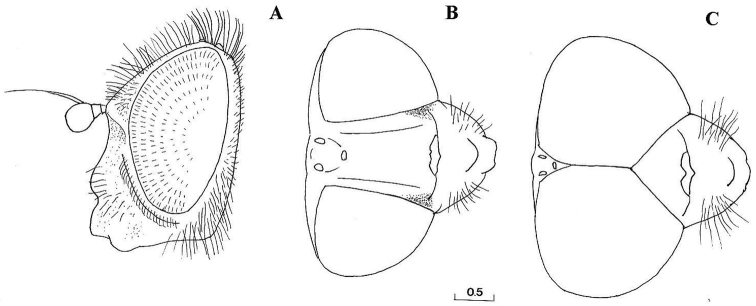
*Cheilosia barbafacies* sp. n., head: **A** female, lateral view **B** female, dorsal view **C** male, dorsal view. Scale in mm.

*Head*: Face with long, predominantly pale hairs, central prominence rounded (Fig. 5); orbital stripe with short, pale hairs. Frontal triangle small, undusted, covered with black hairs; eye contiguity longer than frontal triangle (Fig. 6C). Eyes completely covered with greyish hairs. Occiput narrow, white-grey dusted. Antennae dark, third antennal segment from dark-brown to reddish; arista bare and short (Fig. 11A). Clypeus dusted.

**Figure 7.**
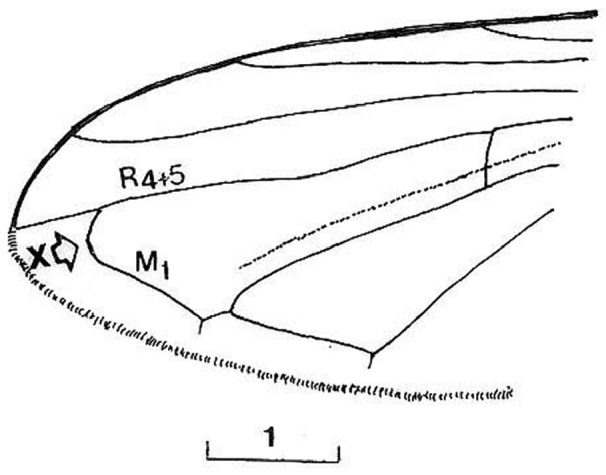
*Cheilosia barbafacies* sp. n., female, tip of wing (x indicates the meeting point of vein M_1_ and vein R_4+5_). Scale in mm.

*Thorax*: Scutum with dark-olive shine, laterally slightly dusted, covered with long, pale and black hairs (Fig. 9A); central disc shining, with fine puncturation. Scutellum covered with long hairs and numerous longer black hairs on posterior margin (Fig. 9A). Pleurae dusted, covered with predominantly pale hairs mixed with black hairs on anepisternum and anepimeron; katepisternum continuosly pilose. *Wing* brownish, with dark veins, completely covered with microtrichia; vein M1 meeting vein R_4+5_ at an obtuse angle (Fig. 7: x). Calypters yellowish-grey. Haltere yellowish-grey with dark capitulum. *Legs* dark, except pale apex of femora, basal 1/3–1/4 and apical 1/5–1/6 of tibiae and ventral surface of tarsi on fore and middle legs; hairs on legs predominately pale mixed with black.

*Abdomen*: Tergites shining, except the whole tergite 2 and dull central area on tergites 2 and 3, which extends from anterior margin of tergite 2 to basal 6/7 of tergite 3, leaving the posterior margin of tergite 3 shining; tergites covered with erected, pale hairs, except few black hairs on posterior half of tergite 4 and on pregenital segments. Sternites grey dusted covered with pale hairs.

**Figure 8.**
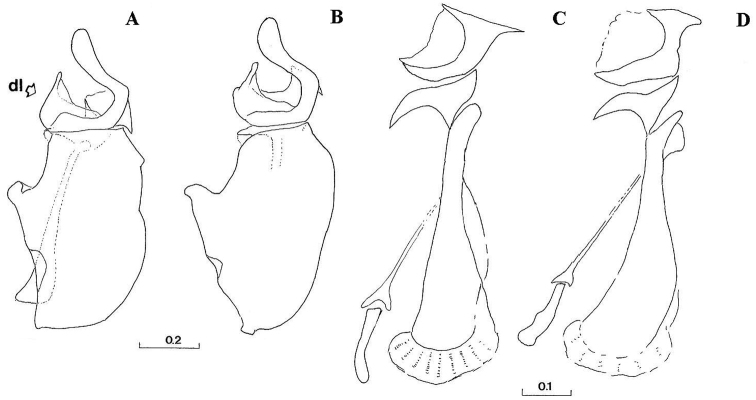
**A–B** Male genitalia, hypandrium, right lateral view (dl indicates the dorsal lobe of gonostylus): **A** *Cheilosia barbafacies* sp. n. **B** *Cheilosia pascuorum*. **C–D** Male genitalia, aedeagus and associated structures, right lateral view: **C** *Cheilosia barbafacies* sp. n. **D** *Cheilosia pascuorum*. Scales in mm.

*Genitalia*: Dorsal lobe of gonostylus broad basally (Fig. 8A), without distinct dorsolateral extension (Figs 1A, 2A, 2B), present in *Cheilosia pascuorum* (Figs 1B, 1C, 2C, 2D); theca of hypandrium in ventral view with large quadrilateral excavation (Figs 4A, 4B).

**Figure 9.**
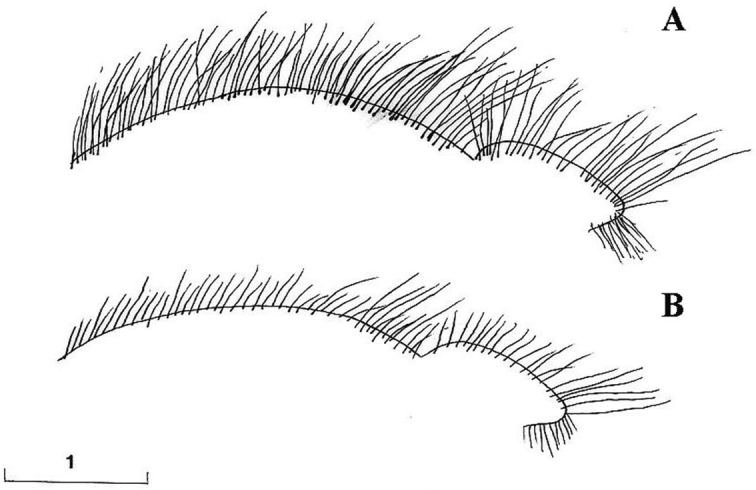
*Cheilosia barbafacies* sp. n., mesonotum, lateral view: **A** male **B** female. Scale in mm.

**Figure 10. F10:**
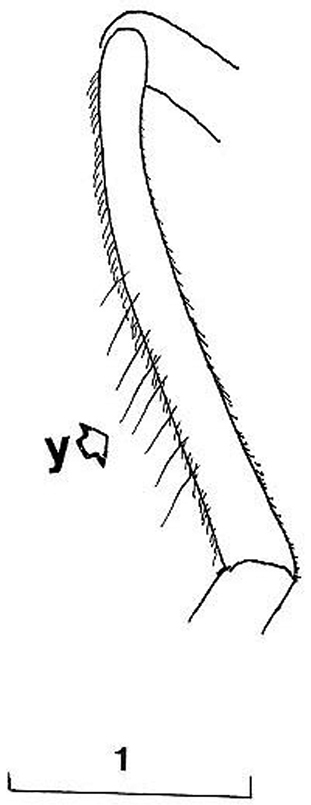
*Cheilosia barbafacies* sp. n., hind tibia, posterodorsal view (y indicates the hairs on posterodorsal surface). Scale in mm.

**Figure 11.**
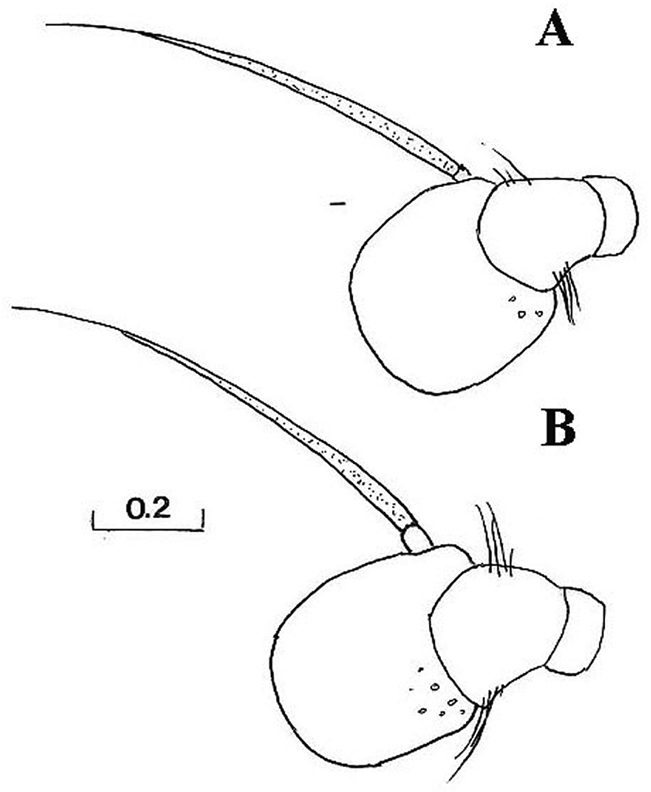
*Cheilosia barbafacies* sp. n., antennae, lateral view: **A** male **B** female. Scale in mm.

FEMALE (Figs 6A, 6B, 7, 9B, 10, 11B). Similar to the male, except for normal sexual dimorphism and the following characters: pile is general shorter and more extensively pale, and legs less dark, basal 1/4 of femora, basal 1/3 and apical 1/4 of tibiae pale; frons with two lateral channels, shiny, except dusted antero-lateral corners (Fig. 6B), covered with pale hairs, except a few black hairs around ocellar triangle and above antennae; thorax pale haired, except for a few black hairs on post-alar calli and near wing base; tibiae of fore and middle legs pale, except dark central ring; basal tarsi of fore and middle legs pale; tergites covered with long and erect hairs, except adpressed hairs on central part of tergites 1–4.

**Size**. Male, body length: 8.9–11.1 mm; wing length: 7.9–9.2 mm (14 specimens were measured). Female, body length: 10.5 mm; wing length: 8.7 mm (1 specimen was measured).

##### Diagnosis.

Species related to *Cheilosia pascuorum*, but differs in the following characteristics: face covered with long hairs (Figs 5, 6), bare in *Cheilosia pascuorum*; clypeus dusted, shining in *Cheilosia pascuorum*; male genitalia: dorsal lobe of gonostylus without distinct dorsolateral extension (Figs 1A, 2A, 2B), present in *Cheilosia pascuorum* (Figs 1B, 1C, 2C, 2D).

Based on the identification keys for European hoverflies, this species can be confused with four other *Cheilosia* species which have long facial hairs: *Cheilosia barbata* (Fig. 3A), *Cheilosia lasiopa* Kowarz, 1885 (Fig. 4D), *Cheilosia melanopa* (Fig. 4E) and *Cheilosia vulpina* (Fig. 4C). Identification of the new described species is possible based on a combination of the following characters: arista bare; central disc of scutum shining; vein M1 meeting vein R_4+5_ at an obtuse angle (Fig. 7: x); tergites 1-3 pale haired; sternites obviously grey dusted; male: dorsal lobe of gonostylus broader basally (Fig. 8A); female: arista about 3 times as long as third antennal segment (Fig. 11B); hairs on scutum long and erected (Fig. 9B); hind tibia on posterodorsal surface with few longer hairs (Fig. 10: y).

##### Etymology.

The specific epithet is derived from the Latin nouns (in apposition) of feminine gender in the nominative case: “barba” (beard) and “facies” (face). The name indicates the presence of long hairs on the face.

##### Distribution

(Fig. 13)**.** *Cheilosia barbafacies* sp. n. is found in two Dinaric mountains in the central part of the Balkan Peninsula, in Durmitor (Montenegro) and Jahorina (Bosnia-Herzegovina), while the related species, *Cheilosia pascuorum*, has a wider range extending from the Alps, across the Balkan Peninsula, to Romania and the European part of Russia (Speight 2012). Both species appear sympatrically within one refuge area rich in endemics and relict species (Durmitor mountain, gorge of river Sušica and glacial lakes Škrčka jezera) (Šimić 1987, Vujić 1996).

##### Discussion.

Although *Cheilosia barbafacies* has long facial hairs, it is closely related to *Cheilosia pascuorum* with a non-hairy face. Species with long facial hairs were assigned to “group B” of Sack (1928-1932), based on Becker’s (1894) revision of the genus. Also recent authors (e.g. Bartsch et al. 2009) still use this old name (“group B”) in their keys for practical reasons. In his recent classification of the genus, Barkalov (2002) included taxa with long facial hairs in seven out of his thirteen subgenera, i.e. subgenera *Cheiloisa* s.str., *Convocheila* Barkalov, 2002, *Endoiasimyia* Bigot, 1882, *Floccocheila* Barkalov, 2002, *Hiatomyia* Shannon, 1922, *Neocheilosia* Barkalov, 1983 and *Taeniochilosia* Oldenberg, 1916. Phylogenetic analysis of the relationship between the genus *Cheilosia* and the tribe Rhingiini, based on mtDNA COI gene sequence (Ståhls et al. 2004), revealed that *Cheilosia* species with this character state can be found in eight clades (see Fig. 1 in Ståhls et al. 2004). It seems clear that the length of the facial pilosity is not a synapomorphic character of any species group, and might evolve several times within the genus *Cheilosia*.

In Bartsch et al. (2009), specimens of *Cheilosia barbafacies* key out to *Cheilosia vulpina* (group B), and in Van Veen (2004), males and females with dark-brown antennae can be identified as *Cheilosia vulpina*, while specimens with reddish antennae are *Cheilosia barbata*. For the separation of *Cheilosia barbafacies* from *Cheilosia barbata*, diagnostic characters of the *proxima* group can be used, and the distinction between *Cheilosia vulpina* and *Cheilosia barbafacies* sp. n. is presented in the following key.

Some females of *Cheilosia barbafacies* sp. n. are similar to females of *Cheilosia redi*, and they can be separated by the following characters:

*Cheilosia barbafacies* sp. n.: vein M1 meeting vein R_4+5_ with an obtuse angle (Fig. 7: x); hairs on scutum and tergites long and erected (Fig. 9B); hind tibia on posterodorsal surface with few longer hairs (Fig. 10: y).

*Cheilosia redi*: vein M1 meeting vein R_4+5_ with an acute angle; hairs on scutum and tergites shorter and significantly adpressed; hind tibia on posterodorsal surface without long hairs.

**Figure 12.**
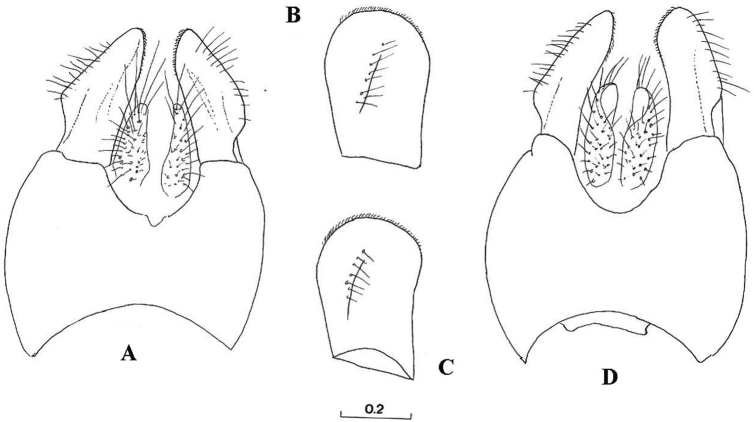
**A, D** Male genitalia, epandrium, dorsal view: **A** *Cheilosia barbafacies* sp. n. **D** *Cheilosia pascuorum* **B, C** Surstylus, right lateral view **B** *Cheilosia barbafacies* sp. n. **C** *Cheilosia pascuorum*. Scale in mm.

**Figure 13.**
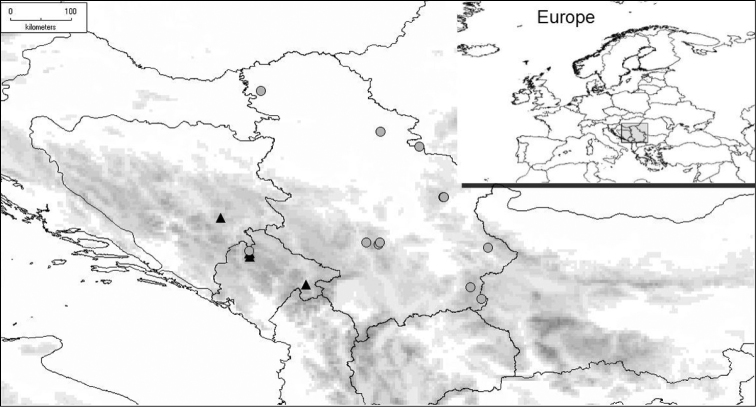
Distribution of *Cheilosia barbafacies* (▲) and *Cheilosia pascuorum* (●).

### Identification key for European species of the *Cheilosia proxima* group

**Table:** 

1	Face with long hairs	2
–	Face bare	3
2	Tergites pale haired (at least 1-3 in males); arista nearly bare. Male: central disc of scutum shining; dorsal lobe of gonostylus broad basally (Fig. 8A). Female: vein M1 meeting vein R_4+5_ at an obtuse angle (Fig. 7: x)	*Cheilosia barbafacies* sp. n.
–	At least tergite 3 in posterior half with black hairs in males, and in females tergites 2-4 with triangular area of adpressed black hairs; arista with short pubescence. Male: central disc of scutum dull; dorsal lobe of gonostylus of a different form (Fig. 4C). Female: vein M1 meeting vein R_4+5_ at right or acute angle	*Cheilosia vulpina* (Meigen, 1822)
3	Holoptic: males	4
–	Dichoptic: females	10
4	3^rd^ antennal segment orange to reddish-brown, at least basoventrally clear reddish	5
–	3^rd^ antennal segment black to blackish-brown (in some specimens paler, but not partly clear reddish)	6
5	Face in lateral view almost flat between central prominence and upper mouth edge; margin of upper calypter often partly with short black setulae; tergite 3 posteromedially with an area of black bristly-hairs, often also tergite 2 with such hairs near hind margin; genitalia (Fig. 3F)	*Cheilosia velutina* Loew, 1840
–	Face in lateral view obviously concave between central prominence and upper mouth edge; margin of upper calypter with pale setulae; tergites 2 and 3 generally with pale (reddish) hairs, but single, short, black-bristly hairs maybe present posteromedially on tergite 3; genitalia (Fig. 3G)	*Cheilosia rufimana* Becker, 1894
6	Margin of upper calypter with short black or dark brown setulae; frons slightly swollen; gonostylus in Fig. 3B	*Cheilosia ingerae* Nielsen & Claussen, 2001
–	Margin of upper calypter with short pale setulae; frons not swollen	7
7	Abdomen (including pregenital segments) pale haired	8
–	Abdomen partly black haired, at least pregenital segments with few black hairs	9
8	Tergite 3 shiny (sometimes dull on anterior margin); vein M1 meeting vein R_4+5_ at an acute angle; arista with short pubescence; dorsal lobe of gonostylus basally narrowed (Fig. 3C)	*Cheilosia balkana* Vujić, 1994
–	Tergite 3 dull centrally; vein M1 meeting vein R_4+5_ at right or obtuse angle; arista bare; dorsal lobe of gonostylus very broad basally (Fig. 8B)	*Cheilosia pascuorum*(Becker, 1894)
9	Basal 2/3 of hind femur with the anterodorsal hair fringe longer than the anteroventral hair fringe; genitalia with the dorsal lobe of gonostylus with a more or less distinct hook on its dorsal margin (Fig. 3D)	*Cheilosia proxima* (Zetterstedt, 1843)
–	Basal 2/3 of hind femur with the anterodorsal hair fringe as long as or shorter than the anteroventral hair fringe; genitalia with the dorsal lobe of gonostylus simple (Fig. 3E)	*Cheilosia gigantea* (Zetterstedt, 1838)
10	3^rd^ antennal segment orange to reddish-brown, at least basoventrally clear reddish	11
–	3^rd^ antennal segment black to blackish-brown (in some specimens paler, but not partly clear reddish)	12
11	Face in lateral view almost flat between central prominence and upper mouth edge; in dorsal view central prominence of face occupying the whole width of face; occiput behind the upper corners of the eyes shining; lunula generally dark or brownish; scutum coarsely punctured, partly wrinkled with short, inclined, pale hairs	*Cheilosia velutina* Loew, 1840
–	Face in lateral view usually concave between central prominence and upper mouth edge (but not distinctly so in all specimens); in dorsal view central prominence of the face not occupying the whole width of the face; occiput behind the upper corners of the eye often completely grey dusted; lunula generally yellowish; scutum with fine punctures, at least anterior half with erect or semi-erect, predominately pale, short hairs, not longer than diameter of hind tibiae, with some longer hairs often intermixed laterally and in front of scutellum	*Cheilosia rufimana* Becker, 1894
12	Frons relatively broad (ratio between length and width 1.2-1.4, average 1.3); pleura: posterior anepisternum predominately shining, at most anterior third of the sclerite thinly dusted; barrette (upper edge of meropleuron) more or less shining, contrasting with the dusting of the adjacent sclerites; basal 2/3 of hind femora with anteroventral hair fringe as long as diameter of hind femur	*Cheilosia ingerae* Nielsen & Claussen, 2001
–	Frons relatively narrow (ratio between length and width 1.4-1.7, average 1.6); generally more than anterior third of posterior anepisternum grey dusted, often sclerite completely dusted; barrette dusted; basal 2/3 of hind femur with or without of anteroventral hair fringe	9
13	Vein M1 meeting vein R_4+5_ at an obtuse angle (as in Fig 7); arista bare (as in Fig. 4B); tergites predominately pale haired	*Cheilosia pascuorum* (Becker, 1894)
–	Vein M1 meeting vein R_4+5_ at an acute or right angle; arista pubescent; tergites partly black haired	14
14	Legs black, exceptionally knees paler	*Cheilosia balkana* Vujić, 1994
–	On legs at least front and mid tibiae pale on both ends	15
15	Basal 2/3 of hind femur with the anteroventral hair fringe long, often obviously longer than diameter of hind femur; apex of hind femur ventrally with some black bristles or spines	*Cheilosia gigantea* (Zetterstedt, 1838)
–	Hind femur without anteroventral hair fringe, occasionally with single longer hairs anteroventrally which are shorter than, or rarely as long as, the diameter of the hind femur; apex of the hind femur ventrally most often without black bristles or spines…	*Cheilosia proxima* (Zetterstedt, 1843)

## Supplementary Material

XML Treatment for Cheilosia barbafacies
